# Effect of differences in O-RADS lexicon interpretation between senior and junior sonologists on O-RADS classification and diagnostic performance

**DOI:** 10.1007/s00432-023-05108-z

**Published:** 2023-07-11

**Authors:** Ya Yang, Hongyan Wang, Zhenzhen Liu, Na Su, Luying Gao, Xixi Tao, Rui Zhang, Yang Gu, Li Ma, Ruojiao Wang, Wen Xu, Yuhuan Xie, Wenjun Zhang, Heng Zhang, Gaiqin Xue, Tong Ru, Qing Dai, Jianchu Li, Yuxin Jiang

**Affiliations:** 1 Department of Ultrasound, Peking Union Medical College Hospital, Chinese Academy of Medical Sciences and Peking Union Medical College, No.1 Shuai Fu Yuan, Dong Cheng District, Beijing, 100730 China; 2 Department of Ultrasound, Dongguan People’s Hospital Affiliated to Southern Medical University, Dongguan, China; 3 Department of Ultrasound, Taihe Hospital, the Affiliated to Hubei University of Medicine, Shiyan, China; 4 Department of Ultrasound, Zhuhai People’s Hospital, Zhuhai, China; 5 Department of Ultrasound, Shanxi Provincial Cancer Hospital, Shanxi, China; 6 Prenatal Diagnosis Center, Drum Tower Hospital, Nanjing University Medical School, Nanjing, China

**Keywords:** Ovarian-adnexal reporting and data system, Adnexal lesions, Ultrasound, Inter-observer agreement, Diagnosis

## Abstract

**Purpose:**

To assess the consistency of Ovarian-Adnexal Reporting and Data System (O-RADS) lexicon interpretation between senior and junior sonologists and to investigate its impact on O-RADS classification and diagnostic performance.

**Methods:**

We prospectively studied 620 patients with adnexal lesions, all of whom underwent transvaginal or transrectal ultrasound performed by a senior sonologist (R1) who selected the O-RADS lexicon description and O-RADS category for the lesion after the examination. Meanwhile, the junior sonologist (R2) analyzed the images retained by R1 and divided the lesion in the same way. Pathological findings were used as a reference standard. kappa (*к*) statistics were used to assess the interobserver agreement.

**Results:**

Of the 620 adnexal lesions, 532 were benign and 88 were malignant. When using the O-RADS lexicon, R1 and R2 had almost perfect agreement regarding lesion category, external contour of solid lesions, presence of papillary inside cystic lesions, and fluid echogenicity (*к*: 0.81–1.00). Substantial agreement in solid components, acoustic shadow, vascularity and O-RADS categories (*к*: 0.61–0.80). Consistency in classifying classic benign lesions in the O-RADS category was only moderate (*к* = 0.535). No significant difference in diagnostic performance between them using O-RADS (P = 0.1211).

**Conclusion:**

There was good agreement between senior and junior sonologists in the interpretation of the O-RADS lexicon and in the classification of O-RADS, except for a moderate agreement in the interpretation and classification of classic benign lesions. Differences in O-RADS category delineation between sonologists had no significant effect on the diagnostic performance of O-RADS.

## Introduction

Ultrasound is the first-line imaging technique for the evaluation of adnexal masses (Vara et al. 2023). However, there is a lack of standardized terminology for gynecologic imaging, which may lead to variation in the interpretation of results between local institutions, and consequently, patients may miss out on the best clinical management strategies (Andreotti et al. 2018). Due to the highly subjective nature of ultrasonography, the subjective assessment of an experienced sonologist is still the most accurate method of gynecologic ultrasonography (Vara et al. 2023; Vázquez-Manjarrez et al. 2020). To improve the consistency and accuracy of ultrasound, the American College of Radiology (ACR) promulgated the Ovarian-Adnexal Reporting and Data System (O-RADS) lexicon in 2018 and built on it with the O-RADS ultrasound risk stratification and management system in 2020 (Andreotti et al. 2018, 2020).

Prior to the formation of the O-RADS committee, the International Ovarian Tumor Analysis (IOTA) group collected decades of outcomes date based on ovarian lesion characteristics, and on this basis published terms and definitions to describe adnexal masses and a series of diagnostic models to assess benign or malignant adnexal masses (Timmerman et al. 2000, 2008, 2016; Van Calster et al. 2014). The O-RADS committee continued some of the terms used by the IOTA group, fully considered the supporting evidence for the terms used to classify benign or malignant lesions and the common use of these terms, reached a consensus of the committee, and ultimately proposed the O-RADS lexicon (Andreotti et al. 2018). The lexicon provides a detailed classification and definition of the main categories of lesions, their size, vascularity, and extra-ovarian findings, as well as specific descriptions of the external and internal features of each of the different categories of lesions (Andreotti et al. 2018, 2020). Based on this lexicon, the experts of the O-RADS working group defined six risk categories for adnexal lesions, category 0 for incomplete evaluation; category 1 for premenopausal normal ovaries; category 2 for almost certainly benign and < 1% risk of malignancy; category 3 for low-risk malignancy (1–10%); category 4 for intermediate risk (10–50%); and category 5 for high risk (≥ 50%) (Andreotti et al. 2020). The classification system also provides corresponding clinical management strategies for different categories of lesions, establishing a bridge between ultrasound and clinical management (Andreotti et al. 2020).

Existing studies (Vara et al. 2022; Xie et al. 2022; Wu et al. 2022; Pi et al. 2021) have confirmed that the O-RADS ultrasound classification system has high sensitivity and diagnostic efficacy when assessed by both senior and junior sonologists, but the inter-observer agreement of this classification system fluctuates widely, ranging from fair to almost perfect. Differences in the interpretation of the O-RADS ultrasound lexicon can have an impact on the outcome of O-RADS assessment (Antil et al. 2022). In order to assess the consistency of O-RADS ultrasound lexicon interpretation between senior and junior sonologists and to investigate its impact on O-RADS classification and diagnostic performance, we conducted a prospective study.

## Materials and methods

The prospective study was approved by the Ethics Committee of our Hospital and registered on ClinicalTrials.gov (ChiCTR2100054542). Informed consent was obtained from all patients who underwent the examination.

### Study participants

**Fig.1:**
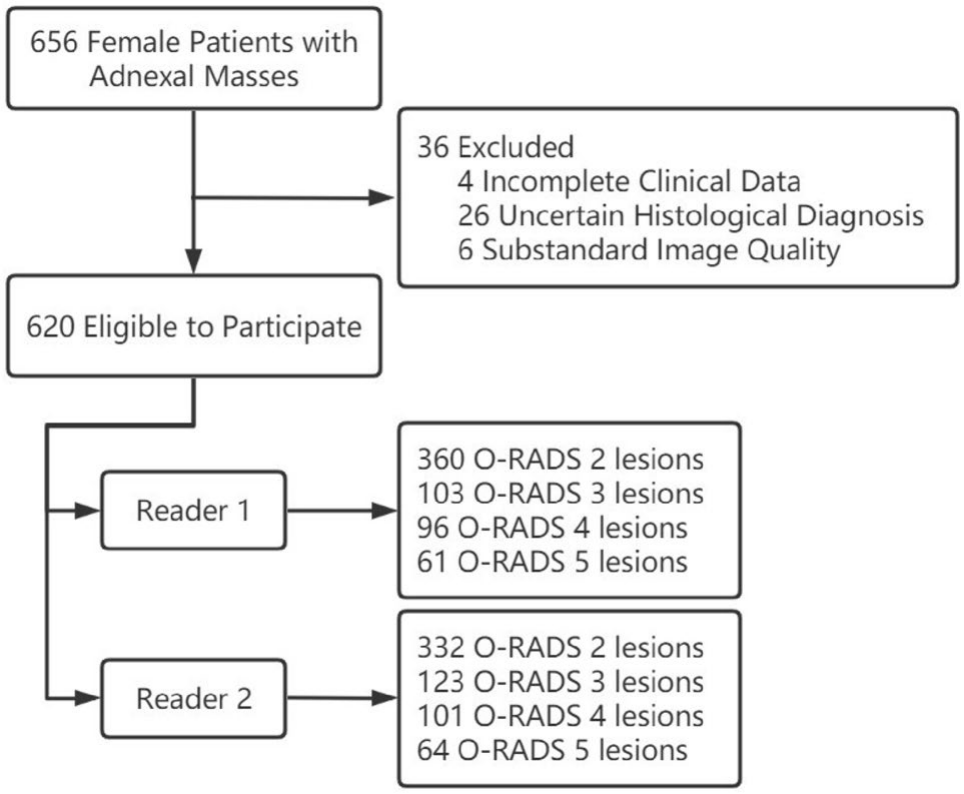
The flow chart of our study. *O-RADS* Ovarian-Adnexal Reporting and Data System

We prospectively studied 656 female patients with adnexal masses between June 2021 and May 2023, and if a patient had multiple adnexal masses (unilateral or bilateral multiple) simultaneously, we selected the lesion with the highest O-RADS category among them for inclusion in the study. The inclusion criteria for this study were preoperative patients with suspicious adnexal lesions detected by clinical palpation or imaging, available pathology and non-malignant postoperative pelvic recurrence. Exclusion criteria were (1) incomplete clinical data of the patients (n = 4), (2) uncertain histological diagnosis (n = 26), and (3) substandard image quality (n = 6). Ultimately, we included 620 adnexal lesions from 620 patients for the study. During the study, we recorded the patients' age, clinical symptoms (abdominal pain, abdominal mass, irregular vaginal bleeding, etc.), age at menarche, menopausal status and CA125 level. The flow chart of our study is shown in Fig. 1.

### Image acquisition and interpretation

All patients underwent transvaginal or transrectal (when patients were unable to undergo transvaginal ultrasound) ultrasound, which was supplemented with transabdominal ultrasound if the lesion was too extensive. The ultrasound instrument used in this study was a Nuewa R9 (Mindray Medical, Shenzhen, China), and all ultrasound images were acquired and interpreted by a sonologist with more than 10 years of experience in gynecologic ultrasound at our institution (Reader 1, R1). This sonologist was studied and trained in the O-RADS ultrasound lexicon and classification system before the start of the study and passed the final test.

During the examination, the sonologist first used B-mode ultrasound to perform a complete scan and evaluation of the lesion, saving images of the largest section of the lesion and its vertical section, and measuring the size of the lesion on the above section. Then, Color Doppler Flow Imaging was performed, and the section of the lesion with the most abundant blood flow was retained. Sections of interest to the sonologist and those with suspicious features of the lesion could also be retained as appropriate. After the examination, the sonologist needed to select the characteristics and the specific O-RADS category of the lesion based on the description of the O-RADS ultrasound lexicon. To assess the differences between senior and junior sonologists when describing the same lesion using the O-RADS ultrasound lexicon, another sonologist with two years of experience in gynecologic ultrasound (Reader 2, R2) who received the same training and examination was selected to analyze the images retained during the examination of R1, and the corresponding features of the lesion and the specific O-RADS category were selected according to the description of the O-RADS ultrasound lexicon. The only patient information available to the two sonologists mentioned above was the patient's age, clinical symptoms, menopausal status, and CA125 level, while the patient's clinical diagnosis and other imaging findings were not available. All images in this study were stored in our hospital's Picture Archiving and Communication Systems (PACS).

### Reference standard

The postoperative pathological findings of the patients were used as a reference standard. All patients included in this study underwent surgery within two weeks of ultrasonography, and all pathologies were classified according to the World Health Organization (WHO) guidelines for the classification of female genital tumors (Meinhold-Heerlein et al. 2016). During the study, borderline tumors (BOT) were categorized as malignant (Basha et al. 2021; Hiett et al. 2022).

### Data analysis

Statistical analyses were performed using SPSS version 21 (IBM Corporation, Armonk, NY) and MedCalc Version 20.022 (MedCalc Software Ltd, Ostend, Belgium) software. Continuous variables were expressed as mean ± standard deviation, and categorical variables were expressed as numbers and percentages. The independent-sample *t* tests were used for the comparison of continuous variables, and chi-square tests were used for the comparison of categorical variables. The kappa value (*к*) was used to assess inter-observer agreement, with *к* equal to or less than 0.20 indicating slight agreement; 0.21–0.40 indicating fair agreement; 0.41–0.60 indicating moderate agreement; 0.61–0.80 indicating substantial agreement; and 0.81–1.00 indicates almost perfect agreement (Landis and Koch 1977). For inter-observer agreement on the internal and external characteristics of solid as well as cystic lesions, we selected both observers classified as the same type of lesion for statistical analysis. The area under the receiver operating characteristic (ROC) curve (AUC) for the classification of benign or malignant tumors was calculated to compare the difference in diagnostic performance between senior and junior sonologists when using O-RADS. When O-RADS > 3 was defined as malignant (Wu et al. 2022; Cao et al. 2021), the O-RADS results were dichotomized accordingly and the sensitivity, specificity, accuracy and positive predictive value (PPV) of O-RADS classification were calculated for both observers by comparison with pathological findings. *P* value < 0.05 was considered statistically significant.

## Results

### Characteristics of patients and lesions

The study ultimately included 620 adnexal lesions in 620 patients, including 532 benign lesions and 88 malignant lesions (including 19 BOT). Of the 620 patients (mean age 39.02 years, range 12–82 years), 510 (82.3%) were non-menopausal women, 42 (6.8%) were early menopausal, and 68 (11.0%) were late menopausal. The median maximum diameter of the lesions was 6.5 cm (range 1.5–25.2 cm). There were significant differences between patients with benign and malignant lesions in age, presence of clinical symptoms, age at menarche, menopausal status, CA 125 level and maximum diameter of the lesions (p < 0.05). The characteristics of the study population and adnexal lesions were detailed in Table 1.

**Table 1:** The characteristics of the study population and adnexal lesions

	Benign, n (%)	Malignant, n (%)	Total	*P*
Age	37.68 ± 11.62	47.08 ± 13.88		< 0.001
Clinical symptoms				< 0.001
No	237 (44.5%)	20 (22.7%)	257	
Yes	295 (55.5%)	68 (77.3%)	363	
Age of menarche	13.30 ± 1.27	14.05 ± 1.83		< 0.001
Menopausal status^*^				< 0.001
Non-menopausal	458 (86.1%)	52 (59.1%)	510	
Early	26 (4.9%)	16 (18.2%)	42	
Late	48 (9.0%)	20 (22.7%)	68	
CA125				< 0.001
Normal	284 (75.5%)	24 (33.8%)	308	
Higher	92 (24.5%)	47 (66.2%)	139	
Maximum diameter	6.82 ± 2.92	9.78 ± 4.43		< 0.001
Position				0.189
Left adnexa	243 (45.7%)	32 (36.4%)	275	
Right adnexa	249 (46.8%)	46 (52.3%)	295	
Others	40 (7.5%)	10 (11.4%)	50	

^*^Early menopausal: postmenopause for < 5 years or ≥ 50 but < 55 years (if uncertain); Late menopausal: postmenopause for ≥ 5 years or ≥ 55 years (if uncertain)

### O-RADS lexicon description of adnexal lesions and inter-observer agreement

Among the 620 adnexal lesions included, the maximum diameter of malignant lesions (9.78 ± 4.43 cm) was significantly larger than that of benign lesions (6.82 ± 2.92 cm) (p < 0.001). When lesions were described using the O-RADS lexicon, the two sonologists had almost perfect agreement (*к*: 0.81–1.00) regarding lesion category, external contour of solid predominant lesions, presence of papillary inside cystic lesions, and fluid echogenicity. Substantial agreement (*к*: 0.61–0.80) in the assessment of the presence or absence of solid components, acoustic shadow and vascularity. However, inter-observer agreement was only moderate (*к* = 0.566) on the assessment of classic benign lesions. The O-RADS lexicon descriptions of adnexal lesions and the inter-observer agreement between the two observers were presented in Table 2.

**Table 2:** The O-RADS lexicon descriptions and inter-observer agreement

	Reader 1		Reader 2		*к* value
	Benign, n (%)	Malignant, n (%)	Benign, n (%)	Malignant, n (%)	
**Lesion category**					0.840
Unilocular, no solid component	343 (100.0%)	0 (0.0%)	322 (99.7%)	1 (0.3%)	
Unilocular cyst with solid component	15 (44.1%)	19 (55.9%)	28 (59.6%)	19 (40.4%)	
Multilocular cyst, no solid elements	131 (97.8%)	3 (2.2%)	127 (96.2%)	5 (3.8%)	
Multilocular cyst with solid component	16 (41.0%)	23 (59.0%)	20 (51.3%)	19 (48.7%)	
Solid or solid appearing	27 (38.6%)	43 (61.4%)	35 (44.3%)	44 (55.7%)	
**solid component**					0.802
Yes	58 (40.6%)	85 (59.4%)	83 (50.3%)	82 (49.7%)	
No	474 (99.4%)	3 (0.6%)	449 (98.7%)	6 (1.3%)	
**Solid or solid-appearing Lesions**					
**External contour**					0.905
Smooth	25 (59.5%)	17 (40.5%)	34 (70.8%)	14 (29.2%)	
Not Smooth	2 (7.1%)	26 (92.9%)	1 (3.2%)	30 (96.8%)	
**Acoustic shadowing**					0.752
Yes	12 (92.3%)	1 (7.7%)	15 (88.2%)	2 (11.8%)	
No	15 (26.3%)	42 (73.7%)	20 (32.3%)	42 (67.7%)	
**Cystic lesion**					
**Papillary**					0.902
Yes	8 (23.5%)	26 (76.5%)	11 (31.4%)	24 (68.6%)	
No	23 (59.0%)	16 (41.0%)	37 (72.5%)	14 (27.5%)	
**Anechoic fluid**					0.991
Yes	153 (91.6%)	14 (8.4%)	154 (92.2%)	13 (7.8%)	
No	352 (91.9%)	31 (8.1%)	343 (91.7%)	31 (8.3%)	
**Classic benign lesions**					0.566
Yes	262 (100.0%)	0 (0.0%)	229 (100.0%)	0 (0.0%)	
No	76 (100.0%)	0 (0.0%)	109 (100.0%)	0 (0.0%)	
**O-RADS categories of Classic benign lesions**					0.535
O-RADS 2	278 (100.0%)	0 (0.0%)	247 (100.0%)	0 (0.0%)	
O-RADS 3	44 (100.0%)	0 (0.0%)	69 (100.0%)	0 (0.0%)	
O-RADS 4	16 (100.0%)	0 (0.0%)	22 (100.0%)	0 (0.0%)	
**Vascularity**					0.804
Color score = 1	377 (99.7%)	1 (0.3%)	335 (99.4%)	2 (0.6%)	
Color score = 2	143 (77.7%)	41 (22.3%)	180 (83.3%)	36 (16.7%)	
Color score = 3	12 (26.1%)	34 (73.9%)	17 (35.4%)	31 (64.6%)	
Color score = 4	0 (0.0%)	12 (100.0%)	0 (0.0%)	19 (100.0%)	

### O-RADS classification results and diagnostic performance of adnexal lesions

**Fig. 2:**
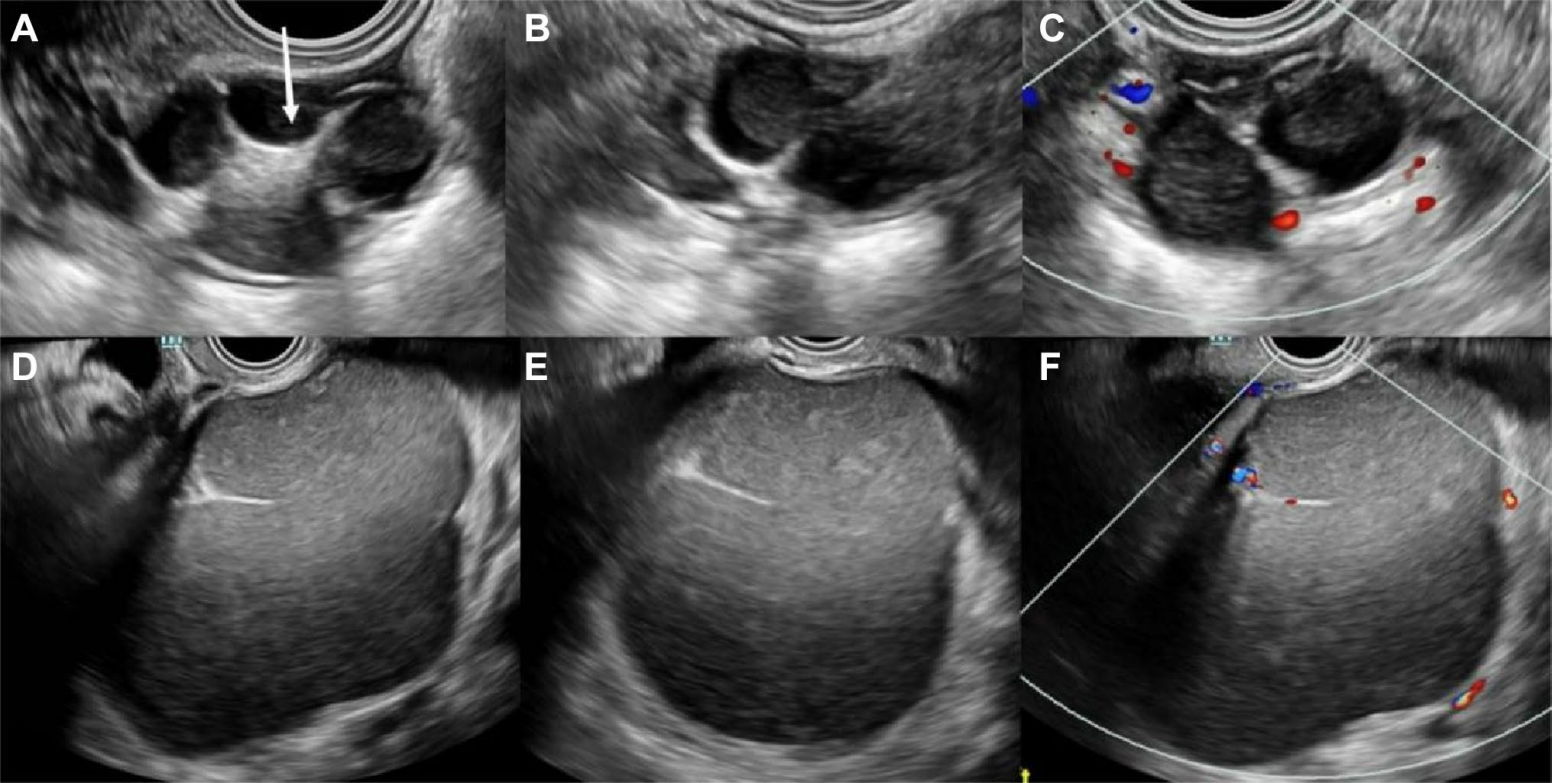
Ultrasound (US) images of classic benign lesions. **A–C** A 38-year-old woman with a right adnexal lesion pathologically confirmed dermoid cyst. **A**, **B** Longitudinal and transverse sections of the lesion. B-mode US showed multilocular cystic lesions, with moderate to high echogenicity in the middle of the lesion (white arrow) and poor sound transmission in the sac. **C** No clear blood flow signal inside the lesion, and a little peripheral blood flow signal (R1: CS = 1; R2: CS = 2). Senior sonologist (R1) classified lesions as classic benign lesion (O-RADS 2) and junior sonologist (R2) as Multilocular cyst with solid component (O-RADS 4). **D–F** A 41-year-old woman with a left adnexal lesion pathologically confirmed endometrioma. **D**, **F**, Longitudinal and transverse sections of the lesion. B-mode US showed multilocular cystic lesions with ground glass echoes. **F** Little blood flow signal in the septation (all CS = 2). R1 classified lesions as classic benign lesion (O-RADS 2) and R2 as multilocular cystic lesions (O-RADS 3)

Table 3 showed the results of O-RADS classification by the two sonologists. In this study, the malignancy rates of lesions classified as O-RADS categories 2, 3, 4, and 5 were 0.0%, 0.0%, 32.3%, and 93.4% (R1) and 0.0%, 2.4%, 24.8%, and 93.8% (R2), respectively, which were consistent with the expected malignancy rates of the lesions. There was substantial agreement between the two observers in classifying O-RADS categories (*к* = 0.790). However, inter-observer agreement on the O-RADS classification of classic benign lesions was only moderate (*к* = 0.535) (Fig. 2).

**Table 3:** O-RADS classification results and inter-observer agreement

		Reader 1			Reader 2			*к* value
		Benign, n (%)	Malignant, n (%)	PPV	Benign, n (%)	Malignant, n (%)	PPV	
O-RADS Score								0.790
**2 [Almost Benign (< 1%)]**				0.0%			0.0%	
Simple cyst (< 10 cm)		76 (21.1%)	0 (0.0%)		77 (23.2%)	0 (0.0%)		
Classic Benign Lesions		257 (71.4%)	0 (0.0%)		231 (69.6%)	0 (0.0%)		
Non-simple unilocular cyst, smooth inner margin (< 10 cm)		27 (7.5%)	0 (0.0%)		24 (7.2%)	0 (0.0%)		
**3 [Low Risk Malignancy (1- < 10%)]**				0.0%			2.4%	
Unilocular cyst ≥ 10 cm		15 (14.6%)	0 (0.0%)		15 (12.5%)	1 (33.3)		
Typical dermoid cysts, endometriomas, hemorrhagic cysts ≥ 10 cm		12 (11.7%)	0 (0.0%)		11 (9.2%)	0		
Unilocular cyst, any size with irregular inner wall < 3 mm height		7 (6.8%)	0 (0.0%)		9 (7.5%)	0		
Multilocular cyst < 10 cm, smooth inner wall, CS = 1–3		60 (58.3%)	0 (0.0%)		69 (57.5%)	2 (66.7%)		
Solid smooth, any size, CS = 1		9 (8.7%)	0 (0.0%)		16 (13.3%)	0		
**4 [Intermediate Risk (10- < 50%)]**				32.3%			24.8%	
Multilocular cyst, no solid component	≥ 10 cm, smooth inner wall, CS = 1–3	18 (27.7%)	1 (3.2%)		19 (25.0%)	3 (12.0%)		
	Any size, smooth inner wall, CS = 4	0 (0.0%)	0 (0.0%)		0 (0.0%)	0 (0.0%)		
	Any size, irregular inner wall and/or irregular septation, any color score	3 (4.6%)	1 (3.2%)		4 (5.3%)	0 (0.0%)		
Unilocular cyst with solid component, any size, 0–3 papillary projections, CS = any		13 (20.0%)	8 (25.8%)		18 (23.7%)	7 (28.0%)		
Multilocular cyst with solid component, any size, CS = 1–2		14 (21.5%)	10 (32.3%)		17 (22.4%)	6 (24.0%)		
Solid smooth, any size, CS = 2–3		17 (26.2%)	11 (35.5%)		18 (23.7%)	9 (36.0%)		
**5 [High Risk (≥ 50%)]**				93.4%			93.8%	
Unilocular cyst, any size, ≥ 4 papillary, CS = any		1 (25.0%)	12 (21.1%)		2 (50%)	13 (21.7%)		
Multilocular cyst with solid component, any size, CS = 3–4		1 (25.0%)	10 (17.5%)		2 (50%)	10 (16.7%)		
Solid smooth, any size, CS = 4		0 (0.0%)	3 (5.3%)		0	4 (6.7%)		
Solid irregular, any size, CS = any		2 (50%)	23 (40.4%)		0	25 (41.7%)		
Ascites and/or peritoneal nodules		0 (0.0%)	9 (15.8%)		0	8 (13.3%)

**Fig. 3:**
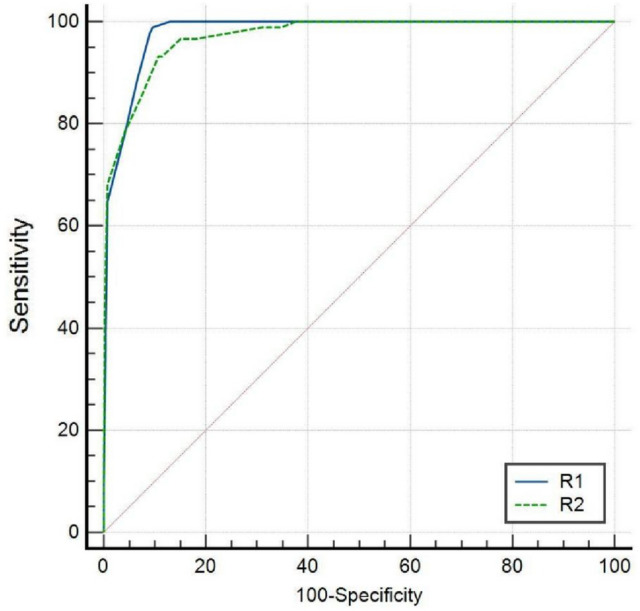
ROC curve of two observers using O-RADS

Defined as malignant when O-RADS > 3, R1 had higher diagnostic sensitivity (100.0% vs 96.6%), specificity (87.0% vs 85.0%), accuracy (88.9% vs 86.6%) and AUC (0.980 vs 0.971) than R2. However, there was no significant difference in diagnostic performance between them (P = 0.1211) (Table 4 and Fig. 3).

**Table 4:** The diagnostic performance of two observers using O-RADS

	Sensitivity (%)	Specificity (%)	Accuracy (%)	PPV	AUC (95% CI)	*P*
Reader 1	100.0%	87.0%	88.9%	56.1%	0.980 (0.965–0.989)	0.1211
Reader 2	96.6%	85.0%	86.6%	51.5%	0.971 (0.954–0.983)

## Discussion

The O-RADS ultrasound working group selected some terms from the O-RADS lexicon and proposed the O-RADS ultrasound risk stratification and management system in 2020 (Andreotti et al. 2020; Cao et al. 2021). This risk stratification and management system provides a detailed description of each category (Andreotti et al. 2020), but in some studies, the classification system still did not achieve good interobserver agreement (Antil et al. 2022; Guo et al. 2022a, 2022b). Differences in the interpretation of the O-RADS ultrasound lexicon can have an impact on the assessment results of O-RADS (Antil et al. 2022). To explore the sources of inter-observer variation in the O-RADS classification system, we analyzed the consistency of the O-RADS ultrasound lexicon applied by senior and junior sonologists to describe the same lesions, and then explored the impact of differences in O-RADS lexicon interpretation on O-RADS classification results and diagnostic performance.

Like the results of Jha et al. (Jha et al. 2022), inter-observer agreement between senior and junior sonologists was almost perfect for lesion type. Inter-observer agreement for blood flow scores was also the same as in previous studies (Substantial agreement) (Antil et al. 2022; Jha et al. 2022). However, the interobserver agreement for the outer contour of the solid component was higher than in previous studies (almost perfect vs moderate) (Antil et al. 2022; Jha et al. 2022), and the reason for this may be that in the present study, the analysis of the external contour of the solid component was performed on the basis of the category in which both observers were classified as solid or solid appearing lesions, and the analysis on this basis may have led to an improved interobserver agreement for this term. In terms of the presence or absence of solid components, inter-observer agreement in this study was poorer than in Jha et al. (2022), but the final agreement was also at a substantial level. In addition, an interobserver agreement analysis of classic benign lesions was included in this study, with only moderate interobserver agreement between the two sonologists in 338 pathologically confirmed classic benign lesions (*к* = 0.566). Senior sonologist was more accurate in identifying and correctly classifying typical benign lesions compared to junior sonologist (77.5% vs. 67.8%).

Consistent with some studies (Basha et al. 2021; Cao et al. 2021; Guo et al. 2022; Katlariwala et al. 2022), the inter-observer consistency of O-RADS classification in this study was substantial (*к* = 0.790), and in this study, O-RADS classification inconsistencies were mostly concentrated in classic benign lesions (*к* = 0.535). When classifying classic benign lesions, junior sonologist preferred to follow the lesion categories in the O-RADS lexicon; therefore, some classic benign lesions were classified by junior sonologist into the subcategories of atypical benign lesions in O-RADS 2 or 3, which in turn affected the overall inter-observer agreement of the O-RADS classification (*к* = 0.790). Numerous studies have confirmed that the best cutoff value for the O-RADS classification system is > O-RADS 3 (Wu et al. 2022; Basha et al. 2021; Cao et al. 2021). Therefore, even when classified according to the lesion categories in the O-RADS ultrasound lexicon, most classic benign lesions could still be accurately categorized as benign. Therefore, this classification system demonstrated high diagnostic performance when assessed by both senior and junior sonologists (AUC: 0.980 and 0.971, p = 0.1211). However, in this study, some classic benign lesions were misclassified into O-RADS 4 categories, resulting in an overestimation of their malignant risk, which in turn affected the diagnostic accuracy of the O-RADS classification system. Among them, the most misclassified lesion type was dermoid cysts. The histological components of dermoid cysts are mixed, which leads to a complex and diverse sonographic presentation (Saida et al. 2021; Saleh et al. 2021). In the present study, the hyperechoic component in some dermoid cysts was easily mistaken for the solid component by junior sonographers, which in turn led to their classification in the higher O-RADS category and overestimation of their risk of malignancy. This may also be one of the reasons why the consistency of observers in the assessment of the presence or absence of a solid component in this study was lower than in previous studies (Jha et al. 2022). Katlariwala et al. (2022) concluded that comparing the lesion echogenicity with the surrounding pelvic or subcutaneous fat echogenicity during evaluation may reduce misclassification between the hyperechoic of dermoid cyst and solid components. In clinical work, the interobserver agreement and diagnostic efficacy of the O-RADS classification and management system may be further improved if the identification of classic benign lesions by the sonologist is enhanced.

The main strength of our study is that we analyzed inter-observer agreement for each subcategory in the O-RADS ultrasound lexicon and the O-RADS classification system separately, based on prospective, large sample data. However, there are still some limitations to this study. First, this study was a single-center study, and the findings still need to be validated in a large-scale multicenter study. Second, the lesions in this study were evaluated by the junior sonologist on the basis of images acquired by the senior sonologist, which may have overestimated inter-observer agreement and the diagnostic performance of the junior sonologist using the O-RADS classification system. Third, the patients included in this study were preoperative, and data on some normal ovaries (O-RADS 1) and non-surgical benign lesions were excluded, which may led to selection bias.

In summary, there was good agreement between senior and junior sonologists in the interpretation of the O-RADS ultrasound lexicon and in the classification of O-RADS categories, except for a moderate observer agreement in the interpretation and classification of classic benign lesions. In addition, differences in O-RADS category delineation among sonologists of different seniority had no significant effect on the diagnostic performance of O-RADS.

## Data Availability

The datasets generated during and/or analysed during the current study are available from the corresponding author on reasonable request.
